# Efficacy of Ingenol Mebutate in the Treatment of Actinic Keratoses: A Pre- and Posttreatment Dermoscopic Comparative Analysis

**DOI:** 10.1155/2018/4381019

**Published:** 2018-08-29

**Authors:** Mattia Carbotti, Rosa Coppola, Salvatore Zanframundo, Valeria Devirgiliis, Vincenzo Panasiti

**Affiliations:** ^1^Laboratory of Microscopic and Ultrastructural Anatomy, Campus Bio-Medico University, Rome, Italy; ^2^Department of Dermatology, Sapienza University, Rome, Italy

## Abstract

Actinic keratosis (AK) is a common skin lesion in adults which usually occurs on chronically photoexposed areas and considered as a precancerous lesion or a superficial squamous-cell carcinoma. Many classifications have been proposed and its diagnosis is generally clinical but, sometimes, its wide variety of presentations can make diagnosis difficult, even among expert observers. The malignant potential of AKs imposes an early diagnosis and treatment in order to reduce morbidity and mortality, and, for the characterization of photodamaged skin, noninvasive diagnostic techniques, such as dermoscopy, have proved to be useful, while multiple therapeutic strategies, lesion-directed versus field-directed therapies, are available for the treatment of AKs. In this study, we evaluated the efficacy of ingenol mebutate for the treatment of AKs, with a particular focus on patients' compliance, correlating it to clinical and dermoscopic grading, pre- and posttreatment, of these lesions. Fifty-two enrolled patients with AKs received treatment with ingenol mebutate gel (0.015% for face and scalp; 0.05% for trunk and extremities) and multiple dermatological evaluations. End points of the study were complete and partial clearance of clinically visible AKs on day 90. All acquired data were recorded and statistical analyses were performed. Univariate and multivariate analyses were used to identify possible predictive factors. We retrospectively analyzed patient-related and lesion-related factors to identify which variables, among age, gender, lesion site, pain, LSR score, and pretreatment clinical and dermoscopic grading, could independently predict the response to ingenol mebutate treatment. Our findings showed that pretreatment dermoscopic grade II represents an independent predictive factor of the efficacy of ingenol mebutate therapy (OR=14.78, 95% CI: 1.83–119.59, P=0.012) and that response rates differ on the basis of the treated anatomical sites (OR=0.16, 95% CI: 0.03–0.85, P=0.031). Data from this study provide evidence that ingenol mebutate gel is an effective treatment for AK, with relative ease of use, short exposure, and rapid resolution of local reactions, benefits contributing to high adherence of this therapy. Moreover, dermoscopic analysis of skin lesions offers more information than clinical evaluation alone and can be helpful in identifying different groups of AKs, thus selecting the adequate therapeutic choice.

## 1. Introduction

Actinic keratosis (AK) is a common skin lesion which usually occurs on photoexposed skin as a consequence of the cumulative exposure to ultraviolet (UV) radiation and it can be considered a precancerous lesion or, as more recently stated, a superficial squamous-cell carcinoma (SCC) [1]. In general population, AK has a high prevalence of 11-25% [2], and these percentages increase in the population over 70 years of age [3–5]. AKs typically present on chronically sun-exposed areas, face, neck, bald scalp, dorsum of the hands, and forearms, as erythematous or reddish brown, scaly macules or papules or hyperkeratotic plaques [3]. They can occur as single and well-defined lesions or as multiple, less defined lesions affecting a larger cutaneous area. Most AKs are asymptomatic; however some may cause pruritus or burning. Main risk factors for the development of AKs are prolonged sun exposure, both occupational and recreational, advanced age, male gender, Fitzpatrick skin phototype I or II, poor use of sunscreens and solar burns before 20 years of age, genetic factors, chronic immunosuppression, geographic location, history of previous skin cancers, and family history of skin cancers [6–8]. Cumulative exposure to nonionizing radiation, especially UV, is the most important risk factor, reason for which AKs are typically observed on photoexposed areas of the skin surface.

Over time, numerous classification schemes have been proposed [9, 10], both clinical and histopathological, which make it possible to distinguish AKs in different variants (e.g., hypertrophic, pigmented, bowenoid, acantholytic, lichenoid, cornu cutaneum, actinic cheilitis, etc.). The widely accepted clinical grading includes grade I (slightly palpable AK, better felt than seen); grade II (moderately thick AK, easily felt and seen); and grade III (very thick and hyperkeratotic AK) [11]. By definition, AK is confined to the epidermis; while when invasion of the underlying dermis occurs, the lesion is called squamous-cell carcinoma (SCC), a condition with metastatic potential. The diagnosis of AK is generally clinical, without the need for histological confirmation. However, it can sometimes be difficult because different skin diseases can mimic AK, such as solar lentigo, seborrheic keratosis, discoid lupus erythematosus, verrucous nevi, verruca vulgaris or flat wart, warty dyskeratoma, keratoacanthoma, and Bowen's disease. Since there are no major differences, from a clinical point of view, between AK and SCC, if the lesion is of considerable size, strongly erythematous, pruritic, ulcerated, or atypical, a biopsy should be performed. Moreover, in people with extensive UV-induced skin damage, noninvasive diagnostic methods, such as dermoscopy [12, 13], optical coherence tomography (OCT) [14], and reflectance confocal microscopy (RCM) [15, 16], can help in the diagnosis of the disease from its initial stages, as well as for its monitoring, evaluation of therapy response and as alternative methods to the repetition of biopsies for the characterization of photodamaged skin.

AK is characterized by a chronic and unpredictable nature and its natural history has three possibilities: regression, stable staying, or progression to invasive disease [17, 18]. The potential progression of AKs therefore imposes the need for treatment of such lesions in order to reduce morbidity and mortality. Multiple therapeutic strategies, lesion-directed and field-directed therapies, are available for the treatment of AKs, which do not ensure complete clearance and absence of recurrences. A further limitation of these therapies is represented by the poor adherence of the patients, especially towards topical treatments, which are not easy to apply and often protracted for long periods.

Based on these observations, in this study, we evaluated the efficacy of ingenol mebutate, diterpene ester derived from the* Euphorbia peplus* plant, for the treatment of actinic keratoses, with particular attention to patients' compliance, and the relationship between treatment efficacy and pretreatment clinical and dermoscopic grading, in order to better guide physicians in the choice of this therapy, in relation to the type of actinic keratosis presented by the patient.

## 2. Materials and Methods

### 2.1. Patient Enrollment

Consecutive patients suffering from actinic keratosis were retrospectively enrolled among those who presented at dermatology clinic of the Plastic and Reconstructive Surgery Unit of Campus Bio-Medico University in Rome, between December 2016 and February 2017, to receive treatment with ingenol mebutate gel (0.015% for face and scalp; 0.05% for trunk and extremities).

Eligibility criteria included an age greater than or equal to 18 years and the presence of at least one clinically visible actinic keratosis. As opposed, patients were excluded from this study if they had received previous treatment with laser therapy or cryotherapy in the same area, if they had a history of ingenol mebutate allergy, or if the target treatment area contained hyperkeratotic lesions, cutaneous horns, or wounds. Additional exclusion criteria were recent use of medications or other treatments that could interfere with evaluation of the treatment area (e.g., topical medications, immunosuppressive therapies, cytotoxic drugs, UVB phototherapy, other therapies for AKs, or oral retinoids).

### 2.2. Therapy Setting

Before starting treatment with ingenol mebutate, patients were adequately made aware of the characteristics of the active ingredient, the mode of administration, and possible adverse effects, referred to as local skin reactions (LSR). All patients provided written informed consent to their participation in the study and allowed clinical and dermoscopic pictures of the selected treatment area to be taken and used. Approval for this study was granted by the ethics committee of Campus Bio-Medico University of Rome.

Therapy involved the self-application of ingenol mebutate gel 0.015% (150 mcg/g) to a 25-cm^2^ contiguous area once daily for 3 consecutive days, for AKs on the face or scalp, and of ingenol mebutate gel 0.05% (500 mcg/g) once daily for 2 consecutive days, for AKs on the trunk or extremities.

The simultaneous application of other topical medications during the treatment period was strictly forbidden.

### 2.3. Lesions Assessment

All enrolled patients underwent dermatological examination at baseline (day 1, clinical diagnosis of AK and start of therapy) and on days 5, 14, 28, 45, 60, and 90. To date, these patients are still on follow-up, with dermatological examination every three months, in order to evaluate the possible relapse of disease or the onset of new lesions to be treated.

For the management of lesion sites, the entire body surface was divided into three sections: face, scalp, and trunk and extremities.

The primary end point of the study was complete clearance of all clinically visible actinic keratoses in the treated area on day 90. As secondary end point of the study, partial clearance, defined as a reduction of 75% or more in the number of clinically visible AKs in the target treatment area, was evaluated on day 90.

All lesions were evaluated both clinically and dermoscopically, giving each of them a clinical and dermoscopic grading. In fact, as described in the literature, AK can be subdivided into three different clinical grades, which correspond to specific dermatoscopic, reflectance confocal microscopic, and histopathologic substrates. In particular, Zalaudek et al. [11, 19], studying AKs using dermoscopy, have defined a dermoscopic grading identifying distinct dermoscopic patterns for each grade: grade I (red pseudonetwork pattern); grade II (erythematous background intermingled by white to yellow, keratotic, and enlarged follicular openings, “strawberry pattern”); and grade III (white-yellow structureless areas).

For each patient, a record containing clinical and dermoscopic pictures of the treated sites was drafted, in order to optimize the monitoring of the therapy. All these lesions were analyzed using a Heine Delta® 20 dermatoscope (magnification ×15) (Heine Optotechnik GmbH & Co. KG, Herrsching, Germany) connected to a Nikon Coolpix 4500® camera equipped with an optical zoom (×4) (Nikon Corporation, Tokyo, Japan).

### 2.4. Side Effects: Local Skin Reactions (LSR)

During examination on days 5 and 14, expected local skin reactions, erythema, flaking or scaling, crusting, swelling, vesiculation or pustulation, and erosion or ulceration, were analyzed and recorded by using a grading scale (LSR_x,y,z,…_), ranging from 0 to 4 for each response, thus leading to define, for each patient, a composite local-skin-response score (LSR_TOT_), the sum of the six individual scores (maximum score, 24).

Furthermore, subjective pain of each patient during treatment was assessed using the visual analogue scale (VAS).

### 2.5. Statistical Analysis

The acquired demographic, clinical, and dermoscopic data were entered into a database and correlated with the response to therapy (poor versus partial/complete response) using *χ*^2^ test and ANOVA test as appropriate. After univariate analysis, significant variables (P<0.10) were included in a multivariate logistic regression model in order to identify potential independent predictors of the efficacy of ingenol mebutate; adjusted odds ratios (OR) and 95% confidence interval (CI) were calculated and a* p* value of less than 0.05 (P<0.05) was considered statistically significant.

All statistical analyses were performed with the use of MedCalc® Statistical Software version 15.2 (MedCalc Software bvba, Ostend, Belgium).

## 3. Results

### 3.1. Study Population

A total of 52 patients were included in the study, 40 (76.9%) males and 12 (23.1%) females, with a mean age of 76.4 ± 6.7 years. All the patients completed the applications of ingenol mebutate gel, as scheduled; the majority, 27 (51.9%), were treated on the face and 21 (40.4%) on the scalp, while 4 (7.7%) patients were treated on trunk and extremities.

### 3.2. Response to Treatment Evaluation

**Figure 1:**
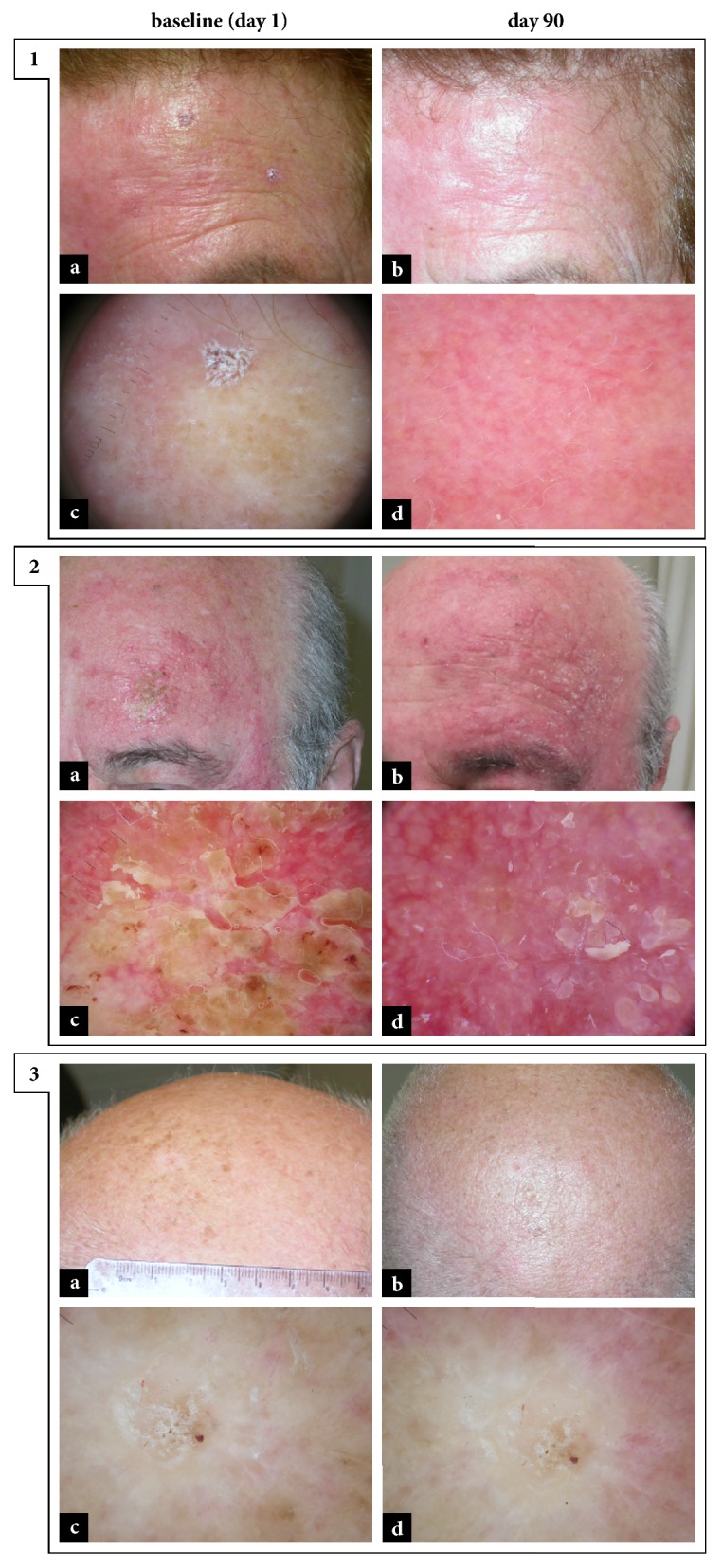
*Ingenol mebutate in the treatment of actinic keratoses.* 1: complete clearance in a patient with AK on left frontal region. Clinical grade decreased from III (a, pretreatment) to 0 (b, posttreatment); dermoscopic grade decreased from III (c, pretreatment) to 0 (d, posttreatment). 2: partial clearance in a patient with AK on left frontotemporal region. Clinical grade decreased from III (a, pretreatment) to I (b, posttreatment); dermoscopic grade decreased from III (c, pretreatment) to II (d, posttreatment). 3: lack of response in a patient with AK on the scalp. Clinical and dermoscopic grade remained stable at III (a-c, pretreatment; b-d, posttreatment).

Regarding efficacy, evaluation of the response to treatment showed that therapy with ingenol mebutate produced a clinical benefit in 84.62% (44/52) of the patients. In particular, at day 90, complete clearance occurred in 25 (48.08%) patients [Figure 1, Panel 1] and partial clearance occurred in 19 (36.54%) patients [Figure 1, Panel 2], while only 8 (15.38%) patients showed poor response [Figure 1, Panel 3]. Specifically, AKs with pretreatment dermoscopic grade II showed a higher rate of complete clearance, 63.64% (7/11), as compared to AKs with pretreatment dermoscopic grade I and III, respectively, 58.33% (7/12) and 24.14% (7/29). Subjects not completely responsive to therapy were subsequently treated by laser therapy until complete resolution.

Furthermore, in order to evaluate which group of actinic keratoses was more responsive to treatment with ingenol mebutate, we compared the mean response-to-therapy values. Specifically, the lesion improvement was quantified by subtracting the value of posttreatment dermoscopic grading to the value of pretreatment dermoscopic grading, for each lesion. In particular, AKs with pretreatment dermoscopic grade II showed a higher mean improvement of the lesion after topical treatment with ingenol mebutate (1.64 ± 0.5, maximum value of 2), as compared to AKs with pretreatment dermoscopic grades I and III, which reported mean improvement values of 0.58 ± 0.51 (maximum value of 1) and 1.72 ± 0.99 (maximum value of 3), respectively (P=0.0001).

There were no significant differences between the proposed groups (poor versus partial/complete response) in basic characteristics such as age and gender or considering VAS and LSR scores. The detailed results of univariate analysis are shown in Table 1.

**Figure 2:**
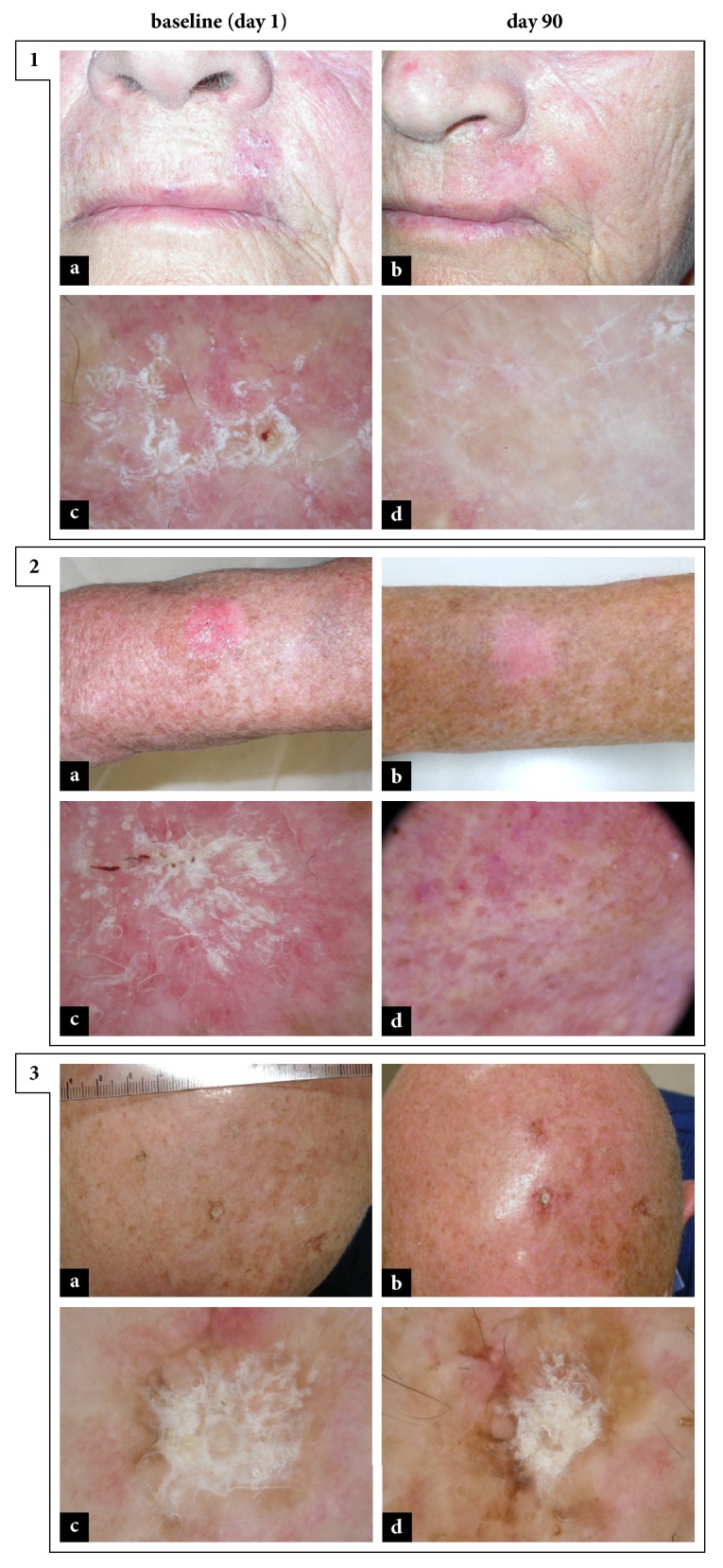
*Different response-to-therapy rates on the basis of the treated anatomical sites.* 1: complete response to therapy in a patient with AK on left upper prolabium. Clinical grade decreased from III (a, pretreatment) to 0 (b, posttreatment); dermoscopic grade decreased from III (c, pretreatment) to 0 (d, posttreatment). 2: complete response to therapy in a patient with AK on left forearm. Clinical grade decreased from III (a, pretreatment) to 0 (b, posttreatment); dermoscopic grade decreased from III (c, pretreatment) to I (d, posttreatment). 3: lack of response to therapy in a patient with AK on the scalp. Clinical and dermoscopic grade remained stable at III (a-c, pretreatment; b-d, posttreatment).

A different rate of effectiveness of topical treatment with ingenol mebutate gel was observed in relation to the site of application. Specifically, at day 90, considering the three macroareas mentioned above, a complete clinical response to the treatment occurred in 66.7% (18/27) of the lesions on the face and in 75% (3/4) of the ones on trunk and extremities, while, regarding the lesions on the scalp, a complete clearance was observed only in 19% (4/21) [Figure 2]. Data just described were even more evident in the context of the analysis of the dermoscopic complete response to treatment. In fact, only 9.5% (2/21) of the lesions on the scalp were completely healed at posttreatment dermoscopic analysis at day 90, compared to 59.3% (16/27) of the lesions on the face and 75% (3/4) of those located on trunk and extremities [Figure 2].

### 3.3. Subjective Pain Assessment

**Figure 3:**
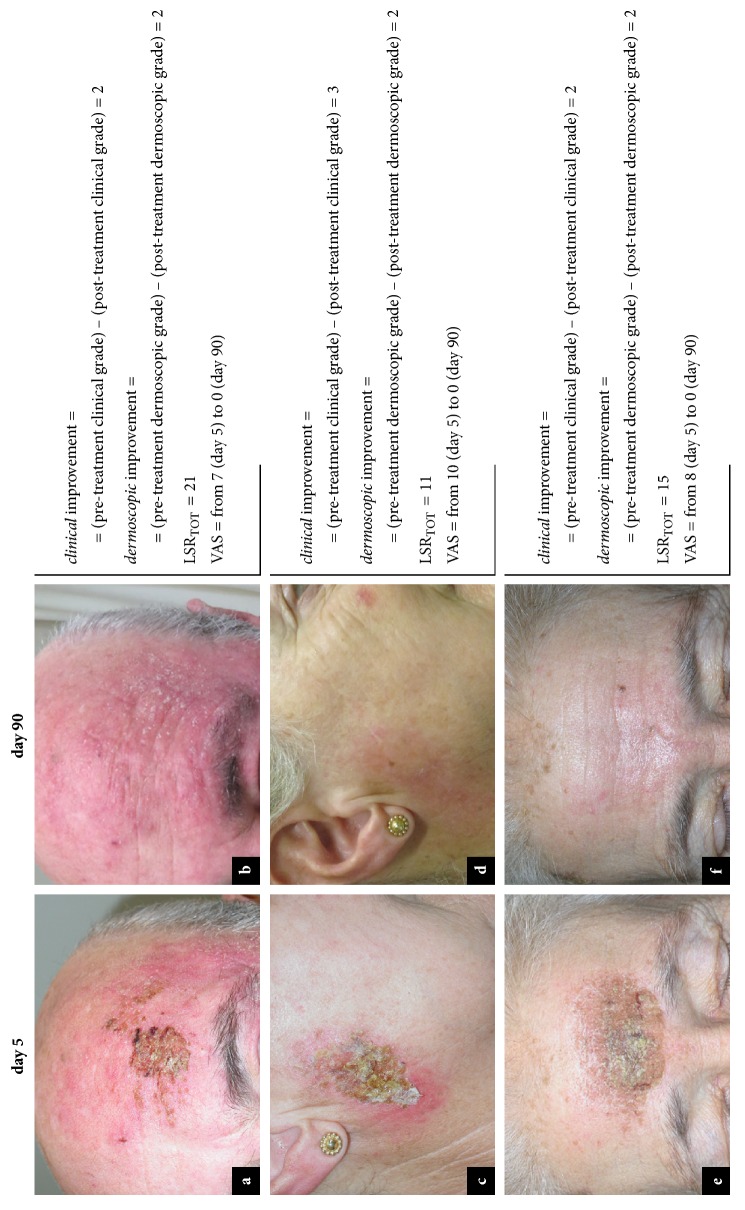
*Local skin reactions, pain during treatment, and clinical and dermoscopic improvement.* Severe local skin reactions (a) and subsequent partial response to therapy (b) in a patient with AK on left frontotemporal region. Mild local skin reactions (c) and subsequent complete response to therapy (d) in a patient with AK on right preauricular region. Severe local skin reactions (e) and subsequent complete response to therapy (f) in a patient with AK on frontal region.

A relationship between the clinical response-to-therapy rate, complete or partial, and the value attributed to the subjective component of pain during treatment, measured by means of VAS scale at day 5, was observed [Figure 3]. Specifically, lesions showing poor clinical response to therapy (n=8) presented a mean value VAS of 2.8 ± 2.4 during treatment, while AKs demonstrating clinical improvement (n=44), complete or partial, showed a mean value VAS of 4.3 ± 2.3 during treatment (P=0.095).

### 3.4. Local Skin Reactions Assessment

All patients presented at least one of the six local skin reactions evaluated. In detail, ordered by decreasing frequency, the following LSRs were observed: erythema, 88% (46/52); flaking or scaling, 67% (35/52); crusting, 58% (30/52); swelling, 52% (27/52); erosion or ulceration, 38% (20/52); and vesiculation or pustulation, 19% (10/52). The mean composite local-skin-response score (LSR_TOT_) for patients treated with ingenol mebutate was 6.2 ± 4 (range 2-21), deriving from the overall analysis of various degrees of severity, from patients with mild reactions (LSR_TOT_ = 2/24) up to a patient who showed more severe reactions (LSR_TOT_ = 21/24) [Figure 3]. Onset of LSRs was analyzed in relation to the poor and complete/partial clinical and dermoscopic regression of lesions. No significant differences were reported (P=0.442, P=0.365).

### 3.5. Multivariate Logistic Regression

Multivariate logistic regression analysis revealed that AKs with a pretreatment dermoscopic grade of II were almost 15 times more likely to achieve a complete clearance (OR=14.78, 95% CI: 1.83–119.59, P=0.012). Conversely, AKs on the scalp were associated with a decreased odd of complete response (OR=0.16, 95% CI: 0.03–0.85, P=0.031), compared to lesions located on the face or trunk and extremities [Table 2]. Other parameters were not demonstrated to be independent predictive factors.

## 4. Discussion

This study aimed to test the efficacy of ingenol mebutate for the therapy of actinic keratoses, correlating it to clinical and dermoscopic grading, pre- and posttreatment, of these lesions. The analysis of our patient cohort showed that ingenol mebutate is effective in the treatment of AKs: at 90 days from the start of treatment, 84.62% (44/52) of patients demonstrated a response, partial or complete, to this therapy. In addition, all patients enrolled, to date still in follow-up, showed no recurrence of disease in the area treated with ingenol mebutate gel. In particular, it should be noted that data presented above were completely compatible with the results of the Phase III study related to this drug [20].

Dermoscopic analysis of skin lesions offers more information than clinical evaluation alone [12, 13], which is why, in this study, we used dermoscopy both in the pretreatment phase and in the phase of evaluation of response to therapy. In fact, lesions that may seem clinically regressed, actually, can still show criteria indicative of the presence of AK, at dermoscopy. To the best of our knowledge, this is the first study to correlate pretreatment dermoscopic grading with the rate of clearance of AKs after therapy with ingenol mebutate, revealing a statistically significant relationship. In particular, pretreatment dermoscopic grading of AK is also correlated with the potential improvement of the lesion after treatment with ingenol mebutate; and this improvement at dermoscopic evaluation, confirmed by multivariate logistic regression analysis, emerged to be superior for grade II AKs (OR=14.78, 95% CI: 1.83–119.59, P=0.012), i.e., those characterized by the so-called “strawberry pattern”. This result could be explained in reason of the mechanism of action of ingenol mebutate: this drug, modulating the inflammatory response, would likely lead to the regression of the erythema observed at dermoscopy. In the context of our series, it also emerged that AKs localized in different anatomical sites present different response to treatment with ingenol mebutate [Figure 2], with excellent rates of complete response obtained at face (59.3-66.7%) and trunk and extremities (75%), percentages that decrease to 9.5-19% in case of lesions on the scalp. This data, confirmed at multivariate logistic regression analysis (lesion site: OR=0.16, 95% CI: 0.03–0.85, P=0.031), could be related to the relative difficulty, for elderly patients (mean age 76.4 ± 6.7, in this study), to apply topical treatment on sites that are difficult to observe independently, thus going to undermine the therapeutic response. Therefore, this type of patients may be advised to be helped in applying the gel on anatomical sites not directly visible and/or accessible, such as the scalp or the back.

The finding that the intensity of subjective pain during treatment with ingenol mebutate was almost double in patients with partial/complete response compared to those with poor response (VAS: 2.8 ± 2.4 versus 4.3 ± 2.3) is of particular interest. In fact, in the first phase of its mechanism of action, the molecule under investigation induces cell death with caustic mechanism and the subsequent locoregional release of proinflammatory cytokines, with consequent recall of cells of innate immunity, generates an inflammatory reaction that, the more intense it is, the more it should cause pain in the patient. Therefore, it could be hypothesized that the intensity of the inflammatory reaction generated by treatment with ingenol mebutate directly correlates with the efficacy of the therapy; further investigations and studies conducted on AKs and SCCs will be necessary to validate this last hypothesis.

Results from this study point out that ingenol mebutate gel, having shown a complete response rate of 48.08%, is an effective treatment for AK. In AKs with pretreatment dermoscopic grade II, the complete clearance rate rises to 63.64%. Moreover, the relative ease of use, short application time and rapidity of resolution of any adverse events, determine a greater adherence to this treatment by patients [21].

Our study has some limitations: first of all, the study was conducted in a single clinical setting and the number of patients was small, which may have limited our ability to discover potentially significant associations. Other limitations were the low number of patients treated on trunk and extremities, the absence of histopathological assessment and correction for multiple testing, the limited number of factors analyzed. Lastly, a major restriction was the retrospective design of the present study. Further larger and controlled studies are needed to confirm our results and to investigate other relevant predictors of the efficacy of ingenol mebutate.

In conclusion, our findings showed that pretreatment dermoscopic grade II represent an independent predictive factor of the efficacy of ingenol mebutate therapy and that response rates differ on the basis of the treated anatomical sites. These results might help clinicians to better select patients affected by AKs to be submitted to treatment with ingenol mebutate.

## Figures and Tables

**Table 1:** Study population and efficacy data, related to clinical and dermoscopic response.

Variable	Clinical response		*P*-value	Dermoscopic response		*P*-value	Total
	Poor	Partial/Complete		Poor	Partial/Complete		
Age	80 ± 4.8	75.7 ± 6.9	0.099	77.7 ± 4.8	76.1 ± 7.1	0.535	76.4 ± 6.7

LSR_TOT_	7.3 ± 2.4	6 ± 4.3	0.442	5.1 ± 2.4	6.5 ± 4.3	0.365	6.2 ± 4

VAS	2.8 ± 2.4	4.3 ± 2.3	0.095	3.6 ± 2.6	4.2 ± 2.4	0.496	4.1 ± 2.4

Gender			0.7522			0.6157	

Male	7 (17.5%)	33 (82.5%)		8 (20%)	32 (80%)		40 (100%)

Female	1 (8.3%)	11 (91.7%)		1 (8.3%)	11 (91.7%)		12 (100%)

Lesion site			0.3245			0.0048	

Face	3 (11.1%)	24 (88.9%)		1 (3.7%)	26 (96.3%)		27 (100%)

Scalp	5 (23.8%)	16 (76.2%)		8 (38.1%)	13 (61.9%)		21 (100%)

Trunk & extremities	0 (0%)	4 (100%)		0 (0%)	4 (100%)		4 (100%)

Pre-treatment clinical grade			0.3802			0.6325	

I	0 (0%)	4 (100%)		0 (0%)	4 (100%)		4 (100%)

II	3 (11.5%)	23 (88.5%)		5 (19.2%)	21 (80.8%)		26 (100%)

III	5 (22.7%)	17 (77.3%)		4 (18.2%)	18 (81.8%)		22 (100%)

Pre-treatment dermoscopic grade			0.2669			0.0232	

I	2 (16.7%)	10 (83.3%)		5 (41.7%)	7 (58.3%)		12 (100%)

II	0 (0%)	11 (100%)		0 (0%)	11 (100%)		11 (100%)

III	6 (20.7%)	23 (79.3%)		4 (13.8%)	25 (86.2%)		29 (100%)

Total n of patients	8 (15.4%)	44 (84.6%)		9 (17.3%)	43 (82.7%)		52 (100%)

LSR, local skin reaction; VAS, visual analogue scale.

**Table 2:** Multivariate logistic regression analysis: factors predicting dermoscopic response to ingenol mebutate therapy.

Variable	OR	95% CI for OR		*P*-value
		Lower	Upper	
Lesion site	0.16	0.03	0.85	0.031

Pre-treatment dermoscopic grade	14.78	1.83	119.59	0.012

OR, odds ratio; CI, confidence interval.

## Data Availability

The data used to support the findings of this study are available from the corresponding author upon request.
